# Ethyl 2-[5-(4-fluoro­phen­yl)pyridin-3-yl]-1-[3-(2-oxopyrrolidin-1-yl)prop­yl]-1*H*-benzimidazole-5-carboxyl­ate

**DOI:** 10.1107/S1600536813014177

**Published:** 2013-06-08

**Authors:** Keng Yoon Yeong, Mohamed Ashraf Ali, Tan Soo Choon, Mohd Mustaqim Rosli, Ibrahim Abdul Razak

**Affiliations:** a Institute for Research in Molecular Medicine, Universiti Sains Malaysia, 11800 USM, Penang, Malaysia; b X-ray Crystallography Unit, School of Physics, Universiti Sains Malaysia, 11800 USM, Penang, Malaysia

## Abstract

In the title compound, C_28_H_27_FN_4_O_3_·H_2_O, the benzimidazole ring system is essentially planar with a maximum deviation of 0.028 (1) Å. It makes dihedral angles of 47.59 (5) and 60.31 (5)°, respectively, with the pyridine and benzene rings, which make a dihedral angle of 22.58 (6)° with each other. The pyrrolidine ring shows an envelope conformation with one of the methyl­ene C atoms as the flap. In the crystal, the components are connected into a tape along the *b-*axis direction through O—H⋯O and O—H⋯N hydrogen bonds and a π–π inter­action between the pyridine and benzene rings [centroid–centroid distance of 3.685 (8) Å]. The tapes are further linked into layers parallel to the *ab* plane by C—H⋯O and C—H⋯F inter­actions.

## Related literature

For biological applications of benzimidazole derivatives, see: Tanious *et al.* (2004 ▶); Coburn *et al.* (1987 ▶); Rao *et al.* (2002 ▶). For a related structure, see: Yoon *et al.* (2012 ▶).
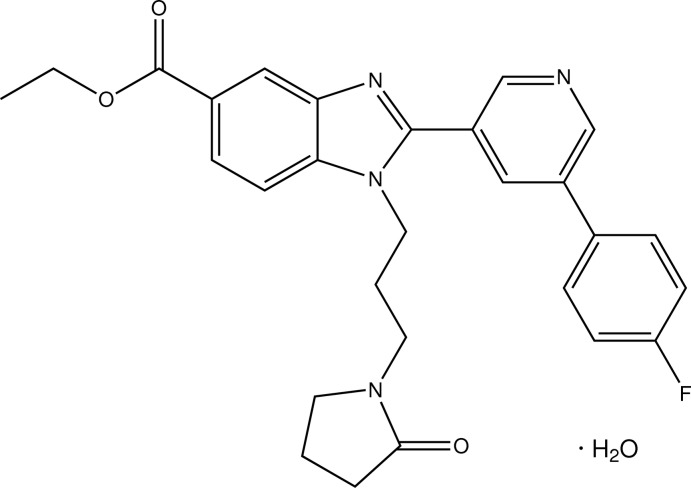


## Experimental

### 

#### Crystal data

C_28_H_27_FN_4_O_3_·H_2_O
*M* *_r_* = 504.55
Monoclinic, 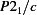

*a* = 16.0640 (15) Å
*b* = 7.6562 (7) Å
*c* = 20.1991 (19) Å
β = 98.163 (2)°
*V* = 2459.1 (4) Å^3^
*Z* = 4
Mo *K*α radiation
μ = 0.10 mm^−1^
*T* = 100 K
0.35 × 0.33 × 0.21 mm

#### Data collection

Bruker APEX Duo CCD area-detector diffractometer
Absorption correction: multi-scan (*SADABS*; Bruker, 2009 ▶) *T*_min_ = 0.967, *T*_max_ = 0.980
24841 measured reflections
6502 independent reflections
5002 reflections with *I* > 2σ(*I*)
*R*_int_ = 0.037

#### Refinement

*R*[*F* ^2^ > 2σ(*F* ^2^)] = 0.042
*wR*(*F* ^2^) = 0.113
*S* = 1.03
6502 reflections
343 parameters
H atoms treated by a mixture of independent and constrained refinement
Δρ_max_ = 0.31 e Å^−3^
Δρ_min_ = −0.25 e Å^−3^

### 

Data collection: *APEX2* (Bruker, 2009 ▶); cell refinement: *SAINT* (Bruker, 2009 ▶); data reduction: *SAINT*; program(s) used to solve structure: *SHELXTL* (Sheldrick, 2008 ▶); program(s) used to refine structure: *SHELXTL*; molecular graphics: *SHELXTL*; software used to prepare material for publication: *SHELXTL* and *PLATON* (Spek, 2009 ▶).

## Supplementary Material

Crystal structure: contains datablock(s) I, New_Global_Publ_Block. DOI: 10.1107/S1600536813014177/is5275sup1.cif

Structure factors: contains datablock(s) I. DOI: 10.1107/S1600536813014177/is5275Isup2.hkl

Supplementary material file. DOI: 10.1107/S1600536813014177/is5275Isup3.cml

Additional supplementary materials:  crystallographic information; 3D view; checkCIF report

## Figures and Tables

**Table 1:** Hydrogen-bond geometry (Å, °)

*D*—H⋯*A*	*D*—H	H⋯*A*	*D*⋯*A*	*D*—H⋯*A*
O1*W*—H1*W*1⋯O3	0.90 (2)	1.89 (2)	2.7881 (16)	174.3 (19)
O1*W*—H2*W*1⋯N2^i^	0.89 (2)	2.04 (2)	2.9309 (16)	175 (2)
C14—H14*A*⋯O1*W* ^ii^	0.95	2.49	3.4368 (17)	173
C17—H17*A*⋯O2^iii^	0.95	2.45	3.1768 (17)	133
C27—H27*A*⋯O2^iv^	0.99	2.45	3.2749 (18)	141
C28—H28*B*⋯F1^i^	0.98	2.43	3.2851 (18)	145

Symmetry codes: (i) 
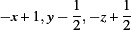
; (ii) 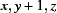; (iii) 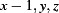; (iv) 
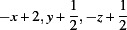
.

**Fig. 1.**
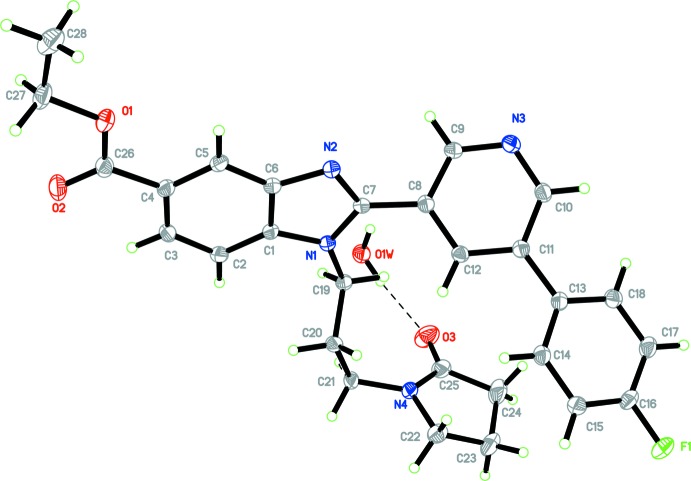
The asymmetric unit of the title compound, showing 50% probability displacement ellipsoids.

**Fig. 2.**
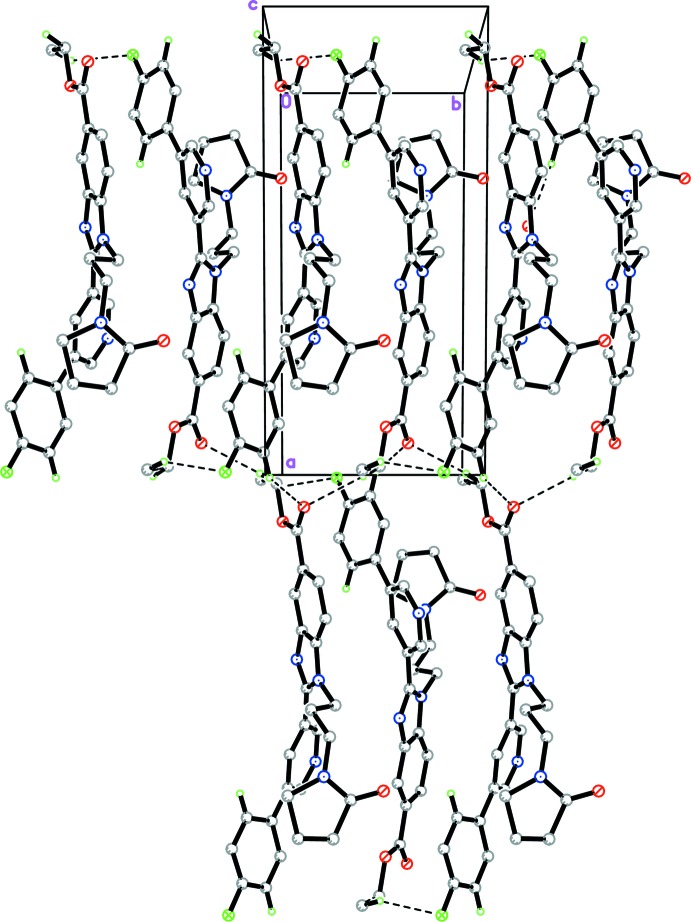
A crystal packing view of the title compound. Dashed lines indicate hydrogen bonds. H atoms not involved in the hydrogen bond interactions have been omitted for clarity.

## supplementary crystallographic information

### Comment

The benzimidazole core is a key building block for many biologically active compounds that play crucial roles in the function of a number of pharmacologically important molecules (Tanious *et al.*, 2004). Substituted benzimidazoles with a wide range of activities such as antibacterial (Coburn *et al.*, 1987) and anti-HIV (Rao *et al.*, 2002) have been reported. As part of our ongoing structural studies of benzimidazole derivatives (Yoon *et al.*, 2012), we now report the structure of the title compound.

In the title compound (Fig. 1), the benzimidazole ring (N1/N2/C1–C7) is planar with a maximum deviation of 0.028 (1) Å for atom N1. It makes dihedral angles of 47.69 (5) and 60.31 (5)°, respectively, with the pyridine (N3/C8–C12) and the benzene (C13–C18) rings. These two rings make a dihedral angle of 22.58 (6)° with each other. The pyrrolidine ring show a sign of envelope conformation with atom C23 at the flap, and with puckering parameters Q = 0.2695 (16) Å and φ = 81.9 (3)°.

In the crystal, the main molecule is connected to the water through O1W—H1W1···O3 and O1W—H2W1···N2^i^ hydrogen bonds (Table 1). The molecules are further linked into a two-dimensional layer parallel to the *ab* plane by C14—H14A···O1W^ii^, C17—H17A···O2^iii^, C27—H27A···O2^iv^ and C28—H28B···F1^v^ interactions (Table 1). A π–π interaction observed between the pyridine (N3/C8–C12) and the benzene (C1–C6) rings with a centroid-centroid distance of 3.685 (8) Å (1 - *x*, 1/2 + *y*, 1/2 - *z*) further stabilizes the crystal structure.

### Experimental

Ethyl 3-amino-4-(3-(2-oxopyrrolidin-1-yl)propylamino)benzoate (0.84 mmol) and sodium metabisulfite adduct of 5-(4-fluorophenyl)nicotinaldehyde (1.68 mmol) were dissolved in DMF. The reaction mixture was reflux at 130 °C for 2 hrs. After completion, the reaction mixture was diluted in Ethyl acetate (20 ml) and washed with water (20 ml). The organic layer was collected, dried over Na_2_SO_4_ and the evaporated *in vacuo* to yield the product. The product was recrystallized from ethyl acetate.

### Refinement

H atom in water molecules were located from difference Fourier maps and freely refined. The remaining H atoms were positioned geometrically and refined using a riding model with with C—H = 0.95–0.99 Å and *U*_iso_(H) = 1.2*U*_eq_(C) or 1.5*U*_eq_(C) for methyl H atoms. A rotating group model was applied to the methyl group.

### Crystal data

**Table:**

C_28_H_27_FN_4_O_3_·H_2_O	*F*(000) = 1064
*M**_r_* = 504.55	*D*_x_ = 1.363 Mg m^−^^3^
Monoclinic, *P*2_1_/*c*	Mo *K*α radiation, λ = 0.71073 Å
Hall symbol: -P 2ybc	Cell parameters from 7659 reflections
*a* = 16.0640 (15) Å	θ = 3.0–30.3°
*b* = 7.6562 (7) Å	µ = 0.10 mm^−^^1^
*c* = 20.1991 (19) Å	*T* = 100 K
β = 98.163 (2)°	Block, brown
*V* = 2459.1 (4) Å^3^	0.35 × 0.33 × 0.21 mm
*Z* = 4	

### Data collection

**Table:**

Bruker APEX Duo CCD area-detector diffractometer	6502 independent reflections
Radiation source: fine-focus sealed tube	5002 reflections with *I* > 2σ(*I*)
Graphite monochromator	*R*_int_ = 0.037
φ and ω scans	θ_max_ = 29.0°, θ_min_ = 1.3°
Absorption correction: multi-scan (*SADABS*; Bruker, 2009)	*h* = −21→21
*T*_min_ = 0.967, *T*_max_ = 0.980	*k* = −8→10
24841 measured reflections	*l* = −25→27

### Refinement

**Table:**

Refinement on *F*^2^	Primary atom site location: structure-invariant direct methods
Least-squares matrix: full	Secondary atom site location: difference Fourier map
*R*[*F*^2^ > 2σ(*F*^2^)] = 0.042	Hydrogen site location: inferred from neighbouring sites
*wR*(*F*^2^) = 0.113	H atoms treated by a mixture of independent and constrained refinement
*S* = 1.03	*w* = 1/[σ^2^(*F*_o_^2^) + (0.0504*P*)^2^ + 0.8415*P*] where *P* = (*F*_o_^2^ + 2*F*_c_^2^)/3
6502 reflections	(Δ/σ)_max_ < 0.001
343 parameters	Δρ_max_ = 0.31 e Å^−^^3^
0 restraints	Δρ_min_ = −0.25 e Å^−^^3^

### Special details

**Table:**

Geometry. All e.s.d.'s (except the e.s.d. in the dihedral angle between two l.s. planes) are estimated using the full covariance matrix. The cell e.s.d.'s are taken into account individually in the estimation of e.s.d.'s in distances, angles and torsion angles; correlations between e.s.d.'s in cell parameters are only used when they are defined by crystal symmetry. An approximate (isotropic) treatment of cell e.s.d.'s is used for estimating e.s.d.'s involving l.s. planes.
Refinement. Refinement of *F*^2^ against ALL reflections. The weighted *R*-factor *wR* and goodness of fit *S* are based on *F*^2^, conventional *R*-factors *R* are based on *F*, with *F* set to zero for negative *F*^2^. The threshold expression of *F*^2^ > σ(*F*^2^) is used only for calculating *R*-factors(gt) *etc*. and is not relevant to the choice of reflections for refinement. *R*-factors based on *F*^2^ are statistically about twice as large as those based on *F*, and *R*- factors based on ALL data will be even larger.

### Fractional atomic coordinates and isotropic or equivalent isotropic displacement parameters (Å^2^)

**Table:**

	*x*	*y*	*z*	*U*_iso_*/*U*_eq_	
F1	0.00660 (5)	1.18506 (11)	0.02501 (4)	0.0277 (2)	
O1	0.89089 (5)	0.94479 (13)	0.30464 (5)	0.0235 (2)	
O2	0.92597 (6)	0.83605 (14)	0.20908 (5)	0.0283 (2)	
O3	0.29289 (7)	0.45959 (15)	0.01770 (6)	0.0368 (3)	
N1	0.52774 (6)	0.76622 (14)	0.16989 (5)	0.0155 (2)	
N2	0.56706 (6)	0.87671 (14)	0.27305 (5)	0.0170 (2)	
N3	0.31735 (7)	0.78858 (16)	0.31867 (6)	0.0205 (2)	
N4	0.32987 (6)	0.73537 (15)	−0.01162 (5)	0.0182 (2)	
C1	0.61471 (7)	0.77837 (16)	0.17809 (6)	0.0160 (2)	
C2	0.67326 (8)	0.73151 (17)	0.13630 (7)	0.0184 (3)	
H2A	0.6568	0.6795	0.0938	0.022*	
C3	0.75670 (8)	0.76529 (17)	0.16049 (7)	0.0193 (3)	
H3A	0.7987	0.7358	0.1337	0.023*	
C4	0.78115 (7)	0.84195 (17)	0.22350 (7)	0.0178 (3)	
C5	0.72238 (7)	0.88486 (17)	0.26539 (7)	0.0170 (2)	
H5A	0.7390	0.9360	0.3081	0.020*	
C6	0.63811 (7)	0.84952 (16)	0.24192 (6)	0.0159 (2)	
C7	0.50329 (7)	0.82625 (16)	0.22826 (6)	0.0156 (2)	
C8	0.41508 (7)	0.83587 (16)	0.24061 (6)	0.0158 (2)	
C9	0.39447 (8)	0.77708 (17)	0.30149 (6)	0.0182 (3)	
H9A	0.4376	0.7260	0.3325	0.022*	
C10	0.25739 (8)	0.85882 (18)	0.27371 (7)	0.0191 (3)	
H10A	0.2025	0.8688	0.2857	0.023*	
C11	0.26951 (7)	0.91840 (16)	0.21053 (6)	0.0160 (2)	
C12	0.35139 (7)	0.90683 (16)	0.19441 (6)	0.0163 (2)	
H12A	0.3634	0.9471	0.1523	0.020*	
C13	0.19944 (7)	0.99304 (16)	0.16271 (6)	0.0166 (2)	
C14	0.21604 (8)	1.10604 (17)	0.11189 (7)	0.0187 (3)	
H14A	0.2726	1.1379	0.1089	0.022*	
C15	0.15166 (8)	1.17301 (18)	0.06552 (7)	0.0203 (3)	
H15A	0.1634	1.2498	0.0311	0.024*	
C16	0.07029 (8)	1.12430 (18)	0.07115 (7)	0.0206 (3)	
C17	0.05031 (8)	1.01864 (18)	0.12132 (7)	0.0223 (3)	
H17A	−0.0066	0.9914	0.1247	0.027*	
C18	0.11554 (8)	0.95205 (18)	0.16728 (7)	0.0201 (3)	
H18A	0.1029	0.8778	0.2022	0.024*	
C19	0.47650 (7)	0.68575 (17)	0.11211 (6)	0.0162 (2)	
H19A	0.4186	0.6689	0.1223	0.019*	
H19B	0.4999	0.5693	0.1040	0.019*	
C20	0.47344 (8)	0.79563 (18)	0.04903 (6)	0.0188 (3)	
H20A	0.5313	0.8123	0.0385	0.023*	
H20B	0.4500	0.9121	0.0569	0.023*	
C21	0.41947 (7)	0.70879 (19)	−0.01050 (7)	0.0206 (3)	
H21A	0.4361	0.7557	−0.0524	0.025*	
H21B	0.4312	0.5818	−0.0093	0.025*	
C22	0.29024 (8)	0.90041 (19)	−0.03556 (7)	0.0236 (3)	
H22A	0.3130	0.9429	−0.0757	0.028*	
H22B	0.2988	0.9913	−0.0004	0.028*	
C23	0.19705 (9)	0.8521 (2)	−0.05240 (8)	0.0296 (3)	
H23A	0.1606	0.9487	−0.0410	0.035*	
H23B	0.1824	0.8238	−0.1005	0.035*	
C24	0.18793 (9)	0.6929 (2)	−0.00927 (9)	0.0352 (4)	
H24A	0.1457	0.6107	−0.0322	0.042*	
H24B	0.1709	0.7270	0.0341	0.042*	
C25	0.27508 (8)	0.6116 (2)	0.00055 (7)	0.0245 (3)	
C26	0.87277 (8)	0.87268 (17)	0.24398 (7)	0.0204 (3)	
C27	0.97999 (8)	0.9758 (2)	0.32679 (8)	0.0273 (3)	
H27A	1.0033	1.0541	0.2950	0.033*	
H27B	1.0113	0.8642	0.3291	0.033*	
C28	0.98829 (9)	1.0583 (2)	0.39429 (8)	0.0316 (3)	
H28A	1.0470	1.0917	0.4084	0.047*	
H28B	0.9707	0.9750	0.4264	0.047*	
H28C	0.9526	1.1625	0.3924	0.047*	
O1W	0.41361 (6)	0.24454 (14)	0.08944 (6)	0.0233 (2)	
H1W1	0.3772 (12)	0.315 (3)	0.0644 (10)	0.043 (5)*	
H2W1	0.4181 (12)	0.291 (3)	0.1303 (12)	0.050 (6)*	

### Atomic displacement parameters (Å^2^)

**Table:**

	*U*^11^	*U*^22^	*U*^33^	*U*^12^	*U*^13^	*U*^23^
F1	0.0204 (4)	0.0310 (5)	0.0289 (5)	0.0040 (3)	−0.0065 (3)	0.0031 (4)
O1	0.0147 (4)	0.0285 (5)	0.0262 (5)	−0.0030 (4)	−0.0004 (4)	−0.0006 (4)
O2	0.0175 (4)	0.0359 (6)	0.0329 (6)	−0.0010 (4)	0.0080 (4)	−0.0008 (5)
O3	0.0320 (6)	0.0305 (6)	0.0450 (7)	−0.0029 (4)	−0.0040 (5)	0.0121 (5)
N1	0.0140 (5)	0.0197 (5)	0.0124 (5)	0.0003 (4)	0.0010 (4)	0.0001 (4)
N2	0.0157 (5)	0.0201 (5)	0.0153 (5)	0.0006 (4)	0.0021 (4)	0.0003 (4)
N3	0.0188 (5)	0.0269 (6)	0.0161 (6)	−0.0010 (4)	0.0030 (4)	0.0006 (5)
N4	0.0148 (5)	0.0238 (6)	0.0155 (5)	0.0014 (4)	0.0005 (4)	−0.0001 (4)
C1	0.0148 (5)	0.0173 (6)	0.0155 (6)	0.0005 (4)	0.0011 (4)	0.0031 (5)
C2	0.0191 (6)	0.0211 (6)	0.0151 (6)	0.0021 (5)	0.0027 (5)	0.0015 (5)
C3	0.0182 (6)	0.0211 (6)	0.0197 (7)	0.0028 (5)	0.0059 (5)	0.0043 (5)
C4	0.0154 (5)	0.0180 (6)	0.0196 (7)	0.0009 (4)	0.0012 (5)	0.0046 (5)
C5	0.0166 (6)	0.0177 (6)	0.0160 (6)	−0.0002 (4)	−0.0006 (4)	0.0016 (5)
C6	0.0161 (5)	0.0163 (6)	0.0153 (6)	0.0008 (4)	0.0020 (4)	0.0022 (5)
C7	0.0163 (5)	0.0168 (6)	0.0135 (6)	0.0011 (4)	0.0017 (4)	0.0022 (5)
C8	0.0150 (5)	0.0178 (6)	0.0147 (6)	−0.0013 (4)	0.0019 (4)	−0.0030 (5)
C9	0.0187 (6)	0.0224 (6)	0.0134 (6)	0.0000 (5)	0.0013 (4)	−0.0006 (5)
C10	0.0161 (6)	0.0238 (6)	0.0181 (6)	−0.0015 (5)	0.0043 (5)	−0.0014 (5)
C11	0.0150 (5)	0.0170 (6)	0.0158 (6)	−0.0014 (4)	0.0014 (4)	−0.0022 (5)
C12	0.0168 (5)	0.0184 (6)	0.0136 (6)	−0.0011 (4)	0.0021 (4)	−0.0008 (5)
C13	0.0151 (5)	0.0188 (6)	0.0157 (6)	−0.0004 (4)	0.0014 (4)	−0.0037 (5)
C14	0.0157 (5)	0.0211 (6)	0.0195 (7)	−0.0008 (4)	0.0030 (5)	−0.0020 (5)
C15	0.0211 (6)	0.0221 (6)	0.0178 (7)	0.0015 (5)	0.0028 (5)	0.0002 (5)
C16	0.0171 (6)	0.0226 (6)	0.0205 (7)	0.0042 (5)	−0.0031 (5)	−0.0027 (5)
C17	0.0146 (5)	0.0266 (7)	0.0253 (7)	−0.0002 (5)	0.0009 (5)	−0.0011 (6)
C18	0.0169 (6)	0.0236 (7)	0.0199 (7)	−0.0019 (5)	0.0026 (5)	0.0006 (5)
C19	0.0162 (5)	0.0188 (6)	0.0131 (6)	−0.0005 (4)	0.0007 (4)	−0.0013 (5)
C20	0.0162 (6)	0.0258 (7)	0.0141 (6)	−0.0028 (5)	0.0007 (4)	0.0010 (5)
C21	0.0143 (5)	0.0335 (7)	0.0140 (6)	0.0016 (5)	0.0020 (4)	−0.0020 (6)
C22	0.0247 (6)	0.0231 (7)	0.0225 (7)	0.0036 (5)	0.0009 (5)	0.0001 (6)
C23	0.0215 (6)	0.0345 (8)	0.0306 (8)	0.0084 (6)	−0.0038 (6)	−0.0057 (7)
C24	0.0166 (6)	0.0444 (9)	0.0438 (10)	−0.0016 (6)	0.0016 (6)	0.0020 (8)
C25	0.0200 (6)	0.0298 (7)	0.0224 (7)	−0.0022 (5)	−0.0016 (5)	0.0020 (6)
C26	0.0171 (6)	0.0187 (6)	0.0257 (7)	−0.0005 (5)	0.0034 (5)	0.0047 (5)
C27	0.0143 (6)	0.0300 (7)	0.0361 (9)	−0.0045 (5)	−0.0017 (5)	0.0020 (6)
C28	0.0267 (7)	0.0277 (8)	0.0370 (9)	−0.0032 (6)	−0.0068 (6)	0.0037 (7)
O1W	0.0215 (5)	0.0276 (5)	0.0209 (5)	−0.0001 (4)	0.0031 (4)	−0.0023 (4)

### Geometric parameters (Å, º)

**Table:**

F1—C16	1.3642 (14)	C13—C18	1.3996 (17)
O1—C26	1.3378 (17)	C14—C15	1.3910 (18)
O1—C27	1.4571 (15)	C14—H14A	0.9500
O2—C26	1.2152 (16)	C15—C16	1.3793 (18)
O3—C25	1.2358 (18)	C15—H15A	0.9500
N1—C7	1.3737 (16)	C16—C17	1.370 (2)
N1—C1	1.3864 (15)	C17—C18	1.3939 (18)
N1—C19	1.4652 (15)	C17—H17A	0.9500
N2—C7	1.3244 (15)	C18—H18A	0.9500
N2—C6	1.3946 (16)	C19—C20	1.5216 (18)
N3—C9	1.3362 (16)	C19—H19A	0.9900
N3—C10	1.3391 (17)	C19—H19B	0.9900
N4—C25	1.3398 (18)	C20—C21	1.5309 (18)
N4—C21	1.4506 (15)	C20—H20A	0.9900
N4—C22	1.4658 (17)	C20—H20B	0.9900
C1—C2	1.3968 (18)	C21—H21A	0.9900
C1—C6	1.4010 (18)	C21—H21B	0.9900
C2—C3	1.3844 (17)	C22—C23	1.5327 (19)
C2—H2A	0.9500	C22—H22A	0.9900
C3—C4	1.4060 (19)	C22—H22B	0.9900
C3—H3A	0.9500	C23—C24	1.517 (2)
C4—C5	1.3934 (18)	C23—H23A	0.9900
C4—C26	1.4899 (17)	C23—H23B	0.9900
C5—C6	1.3959 (16)	C24—C25	1.519 (2)
C5—H5A	0.9500	C24—H24A	0.9900
C7—C8	1.4751 (16)	C24—H24B	0.9900
C8—C9	1.3928 (18)	C27—C28	1.491 (2)
C8—C12	1.3934 (17)	C27—H27A	0.9900
C9—H9A	0.9500	C27—H27B	0.9900
C10—C11	1.3947 (18)	C28—H28A	0.9800
C10—H10A	0.9500	C28—H28B	0.9800
C11—C12	1.4021 (17)	C28—H28C	0.9800
C11—C13	1.4891 (17)	O1W—H1W1	0.90 (2)
C12—H12A	0.9500	O1W—H2W1	0.89 (2)
C13—C14	1.3968 (19)		
			
C26—O1—C27	115.15 (11)	C18—C17—H17A	120.8
C7—N1—C1	106.33 (10)	C17—C18—C13	120.93 (13)
C7—N1—C19	128.87 (10)	C17—C18—H18A	119.5
C1—N1—C19	124.47 (10)	C13—C18—H18A	119.5
C7—N2—C6	104.58 (10)	N1—C19—C20	112.47 (10)
C9—N3—C10	117.03 (12)	N1—C19—H19A	109.1
C25—N4—C21	125.03 (12)	C20—C19—H19A	109.1
C25—N4—C22	113.87 (11)	N1—C19—H19B	109.1
C21—N4—C22	120.64 (11)	C20—C19—H19B	109.1
N1—C1—C2	131.62 (12)	H19A—C19—H19B	107.8
N1—C1—C6	105.67 (11)	C19—C20—C21	111.41 (11)
C2—C1—C6	122.71 (11)	C19—C20—H20A	109.3
C3—C2—C1	116.11 (12)	C21—C20—H20A	109.3
C3—C2—H2A	121.9	C19—C20—H20B	109.3
C1—C2—H2A	121.9	C21—C20—H20B	109.3
C2—C3—C4	121.97 (12)	H20A—C20—H20B	108.0
C2—C3—H3A	119.0	N4—C21—C20	113.66 (11)
C4—C3—H3A	119.0	N4—C21—H21A	108.8
C5—C4—C3	121.45 (11)	C20—C21—H21A	108.8
C5—C4—C26	121.46 (12)	N4—C21—H21B	108.8
C3—C4—C26	117.08 (11)	C20—C21—H21B	108.8
C4—C5—C6	117.14 (12)	H21A—C21—H21B	107.7
C4—C5—H5A	121.4	N4—C22—C23	103.29 (11)
C6—C5—H5A	121.4	N4—C22—H22A	111.1
N2—C6—C5	129.37 (12)	C23—C22—H22A	111.1
N2—C6—C1	110.07 (10)	N4—C22—H22B	111.1
C5—C6—C1	120.55 (11)	C23—C22—H22B	111.1
N2—C7—N1	113.34 (10)	H22A—C22—H22B	109.1
N2—C7—C8	122.57 (11)	C24—C23—C22	103.74 (11)
N1—C7—C8	124.09 (11)	C24—C23—H23A	111.0
C9—C8—C12	118.26 (11)	C22—C23—H23A	111.0
C9—C8—C7	119.18 (11)	C24—C23—H23B	111.0
C12—C8—C7	122.53 (11)	C22—C23—H23B	111.0
N3—C9—C8	123.60 (12)	H23A—C23—H23B	109.0
N3—C9—H9A	118.2	C23—C24—C25	104.00 (12)
C8—C9—H9A	118.2	C23—C24—H24A	111.0
N3—C10—C11	124.86 (11)	C25—C24—H24A	111.0
N3—C10—H10A	117.6	C23—C24—H24B	111.0
C11—C10—H10A	117.6	C25—C24—H24B	111.0
C10—C11—C12	116.70 (11)	H24A—C24—H24B	109.0
C10—C11—C13	121.88 (11)	O3—C25—N4	125.84 (13)
C12—C11—C13	121.41 (11)	O3—C25—C24	126.49 (13)
C8—C12—C11	119.53 (12)	N4—C25—C24	107.66 (13)
C8—C12—H12A	120.2	O2—C26—O1	123.17 (12)
C11—C12—H12A	120.2	O2—C26—C4	123.42 (13)
C14—C13—C18	118.25 (11)	O1—C26—C4	113.41 (11)
C14—C13—C11	120.58 (11)	O1—C27—C28	107.83 (12)
C18—C13—C11	121.16 (12)	O1—C27—H27A	110.1
C15—C14—C13	121.46 (11)	C28—C27—H27A	110.1
C15—C14—H14A	119.3	O1—C27—H27B	110.1
C13—C14—H14A	119.3	C28—C27—H27B	110.1
C16—C15—C14	117.87 (13)	H27A—C27—H27B	108.5
C16—C15—H15A	121.1	C27—C28—H28A	109.5
C14—C15—H15A	121.1	C27—C28—H28B	109.5
F1—C16—C17	118.35 (11)	H28A—C28—H28B	109.5
F1—C16—C15	118.63 (12)	C27—C28—H28C	109.5
C17—C16—C15	123.02 (12)	H28A—C28—H28C	109.5
C16—C17—C18	118.40 (12)	H28B—C28—H28C	109.5
C16—C17—H17A	120.8	H1W1—O1W—H2W1	104.2 (18)
			
C7—N1—C1—C2	177.83 (13)	C13—C11—C12—C8	−179.92 (11)
C19—N1—C1—C2	3.9 (2)	C10—C11—C13—C14	156.88 (13)
C7—N1—C1—C6	−1.04 (13)	C12—C11—C13—C14	−22.24 (18)
C19—N1—C1—C6	−174.99 (11)	C10—C11—C13—C18	−23.22 (19)
N1—C1—C2—C3	178.99 (13)	C12—C11—C13—C18	157.65 (13)
C6—C1—C2—C3	−2.30 (19)	C18—C13—C14—C15	−1.8 (2)
C1—C2—C3—C4	0.13 (19)	C11—C13—C14—C15	178.05 (12)
C2—C3—C4—C5	1.2 (2)	C13—C14—C15—C16	0.0 (2)
C2—C3—C4—C26	−179.73 (12)	C14—C15—C16—F1	−178.27 (12)
C3—C4—C5—C6	−0.41 (19)	C14—C15—C16—C17	2.3 (2)
C26—C4—C5—C6	−179.42 (11)	F1—C16—C17—C18	178.03 (12)
C7—N2—C6—C5	177.95 (13)	C15—C16—C17—C18	−2.5 (2)
C7—N2—C6—C1	−1.19 (14)	C16—C17—C18—C13	0.5 (2)
C4—C5—C6—N2	179.24 (12)	C14—C13—C18—C17	1.6 (2)
C4—C5—C6—C1	−1.70 (18)	C11—C13—C18—C17	−178.31 (12)
N1—C1—C6—N2	1.40 (14)	C7—N1—C19—C20	115.93 (14)
C2—C1—C6—N2	−177.59 (12)	C1—N1—C19—C20	−71.54 (15)
N1—C1—C6—C5	−177.83 (11)	N1—C19—C20—C21	−179.93 (10)
C2—C1—C6—C5	3.18 (19)	C25—N4—C21—C20	−109.17 (15)
C6—N2—C7—N1	0.52 (14)	C22—N4—C21—C20	79.12 (15)
C6—N2—C7—C8	−179.04 (11)	C19—C20—C21—N4	80.43 (14)
C1—N1—C7—N2	0.34 (14)	C25—N4—C22—C23	−12.33 (16)
C19—N1—C7—N2	173.93 (12)	C21—N4—C22—C23	160.26 (12)
C1—N1—C7—C8	179.89 (11)	N4—C22—C23—C24	23.99 (15)
C19—N1—C7—C8	−6.5 (2)	C22—C23—C24—C25	−26.92 (16)
N2—C7—C8—C9	−45.73 (18)	C21—N4—C25—O3	3.8 (2)
N1—C7—C8—C9	134.76 (13)	C22—N4—C25—O3	176.05 (14)
N2—C7—C8—C12	132.21 (13)	C21—N4—C25—C24	−177.20 (12)
N1—C7—C8—C12	−47.30 (19)	C22—N4—C25—C24	−4.99 (17)
C10—N3—C9—C8	0.9 (2)	C23—C24—C25—O3	−160.64 (15)
C12—C8—C9—N3	−1.57 (19)	C23—C24—C25—N4	20.41 (17)
C7—C8—C9—N3	176.45 (12)	C27—O1—C26—O2	−0.48 (19)
C9—N3—C10—C11	0.8 (2)	C27—O1—C26—C4	179.56 (11)
N3—C10—C11—C12	−1.7 (2)	C5—C4—C26—O2	179.52 (13)
N3—C10—C11—C13	179.17 (12)	C3—C4—C26—O2	0.5 (2)
C9—C8—C12—C11	0.57 (18)	C5—C4—C26—O1	−0.52 (18)
C7—C8—C12—C11	−177.39 (11)	C3—C4—C26—O1	−179.57 (11)
C10—C11—C12—C8	0.91 (18)	C26—O1—C27—C28	179.18 (12)

### Hydrogen-bond geometry (Å, º)

**Table:**

*D*—H···*A*	*D*—H	H···*A*	*D*···*A*	*D*—H···*A*
O1*W*—H1*W*1···O3	0.90 (2)	1.89 (2)	2.7881 (16)	174.3 (19)
O1*W*—H2*W*1···N2^i^	0.89 (2)	2.04 (2)	2.9309 (16)	175 (2)
C14—H14*A*···O1*W*^ii^	0.95	2.49	3.4368 (17)	173
C17—H17*A*···O2^iii^	0.95	2.45	3.1768 (17)	133
C27—H27*A*···O2^iv^	0.99	2.45	3.2749 (18)	141
C28—H28*B*···F1^i^	0.98	2.43	3.2851 (18)	145

Symmetry codes: (i) −*x*+1, *y*−1/2, −*z*+1/2; (ii) *x*, *y*+1, *z*; (iii) *x*−1, *y*, *z*; (iv) −*x*+2, *y*+1/2, −*z*+1/2.
